# Correction: Morphological and molecular identification of the dioecious “African species *Volvox rousseletii* (Chlorophyceae) in the water column of a Japanese lake based on field-collected and cultured materials

**DOI:** 10.1371/journal.pone.0223006

**Published:** 2019-09-19

**Authors:** Ryosuke Kimbara, Nanako Isaka, Ryo Matsuzaki, Hiroko Kawai-Toyooka, Masanobu Kawachi, Hisayoshi Nozaki

In the title of this article, there is a missing closing quotation mark after ‘African species.’ The correct title is: Morphological and molecular identification of the dioecious “African species” *Volvox rousseletii* (Chlorophyceae) in the water column of a Japanese lake based on field-collected and cultured materials.

The correct citation is: Kimbara R, Isaka N, Matsuzaki R, Kawai-Toyooka H, Kawachi M, Nozaki H (2019) Morphological and molecular identification of the dioecious “African species” *Volvox rousseletii* (Chlorophyceae) in the water column of a Japanese lake based on field-collected and cultured materials. PLoS ONE 14(8): e0221632. https://doi.org/10.1371/journal.pone.0221632
