# Supplementary material for: Morphological and molecular identification of the dioecious “African species Volvox rousseletii (Chlorophyceae) in the water column of a Japanese lake based on field-collected and cultured materials
Source: PLoS One. 2019 Aug 29;14(8):e0221632. doi: 10.1371/journal.pone.0221632 (PMC6715204; doi:10.1371/journal.pone.0221632)
Supplement: S1 Fig — (DOCX) [file pone.0221632.s001.docx]

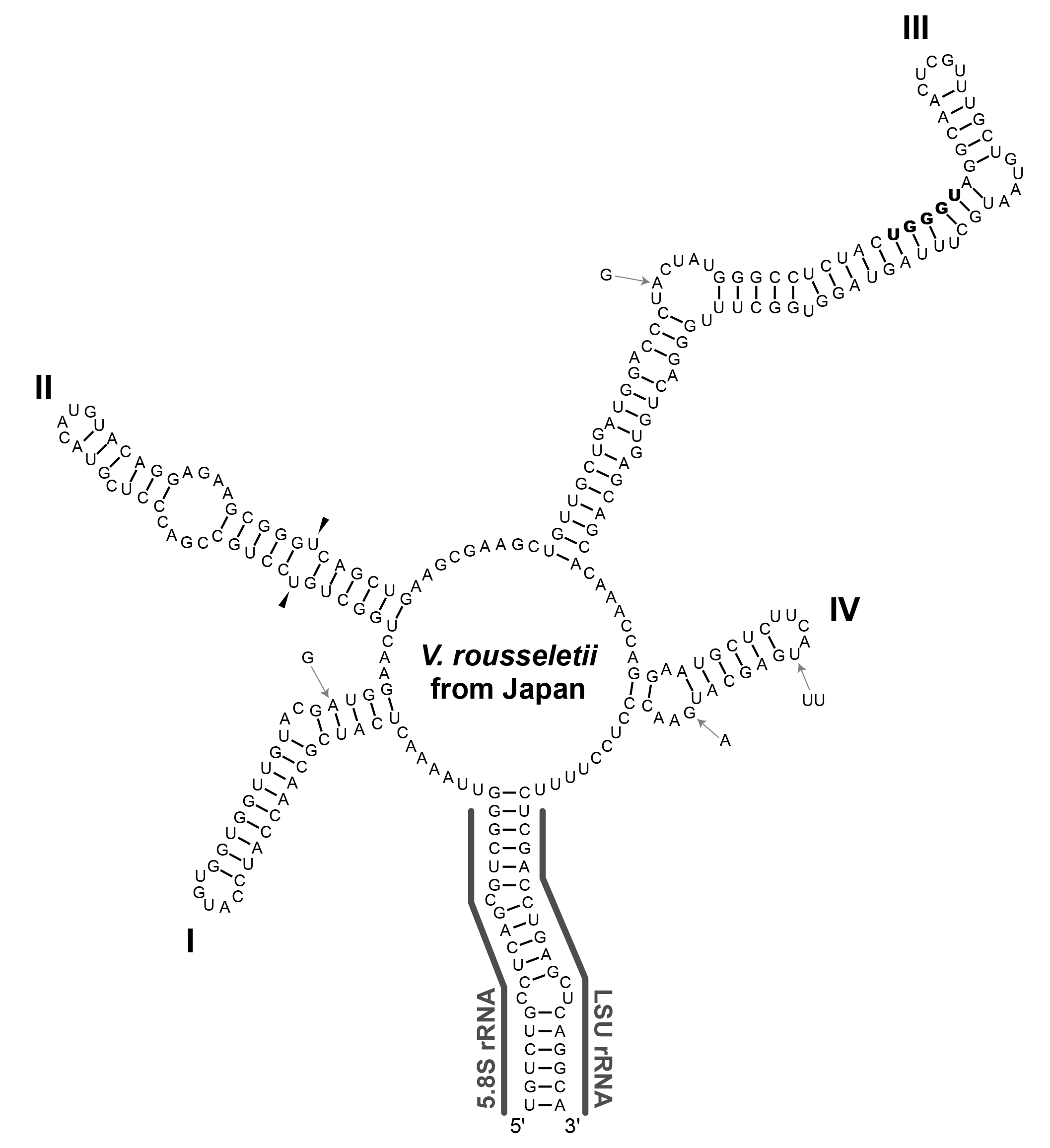


**S1 Fig. The secondary structure of nuclear ribosomal DNA (rDNA) internal transcribed spacer 2 (ITS-2) transcript of Volvox rousseletii from Japan, including the 3’ end of the 5.8S ribosomal RNA (rRNA) and the 5’ end of the large subunit of rRNA (LSU rRNA).**

Secondary structure of nuclear rDNA ITS-2 was drawn using VARNA version 3.9. Note the U-U mismatch in helix II (arrowheads) and the YGGY motif (UGGGU) on the 5’ side near the apex of helix III (boldface), common structural hallmarks of eukaryotic nuclear rDNA ITS-2 secondary structures. Differences within *V. rousseletii* (vs. UTEX 1862 from South Africa) are shown by characters just outside the secondary structure.
