# Supplementary material for: Morphological and molecular identification of the dioecious “African species Volvox rousseletii (Chlorophyceae) in the water column of a Japanese lake based on field-collected and cultured materials
Source: PLoS One. 2019 Aug 29;14(8):e0221632. doi: 10.1371/journal.pone.0221632 (PMC6715204; doi:10.1371/journal.pone.0221632)
Supplement: S2 Fig — (DOCX) [file pone.0221632.s002.docx]

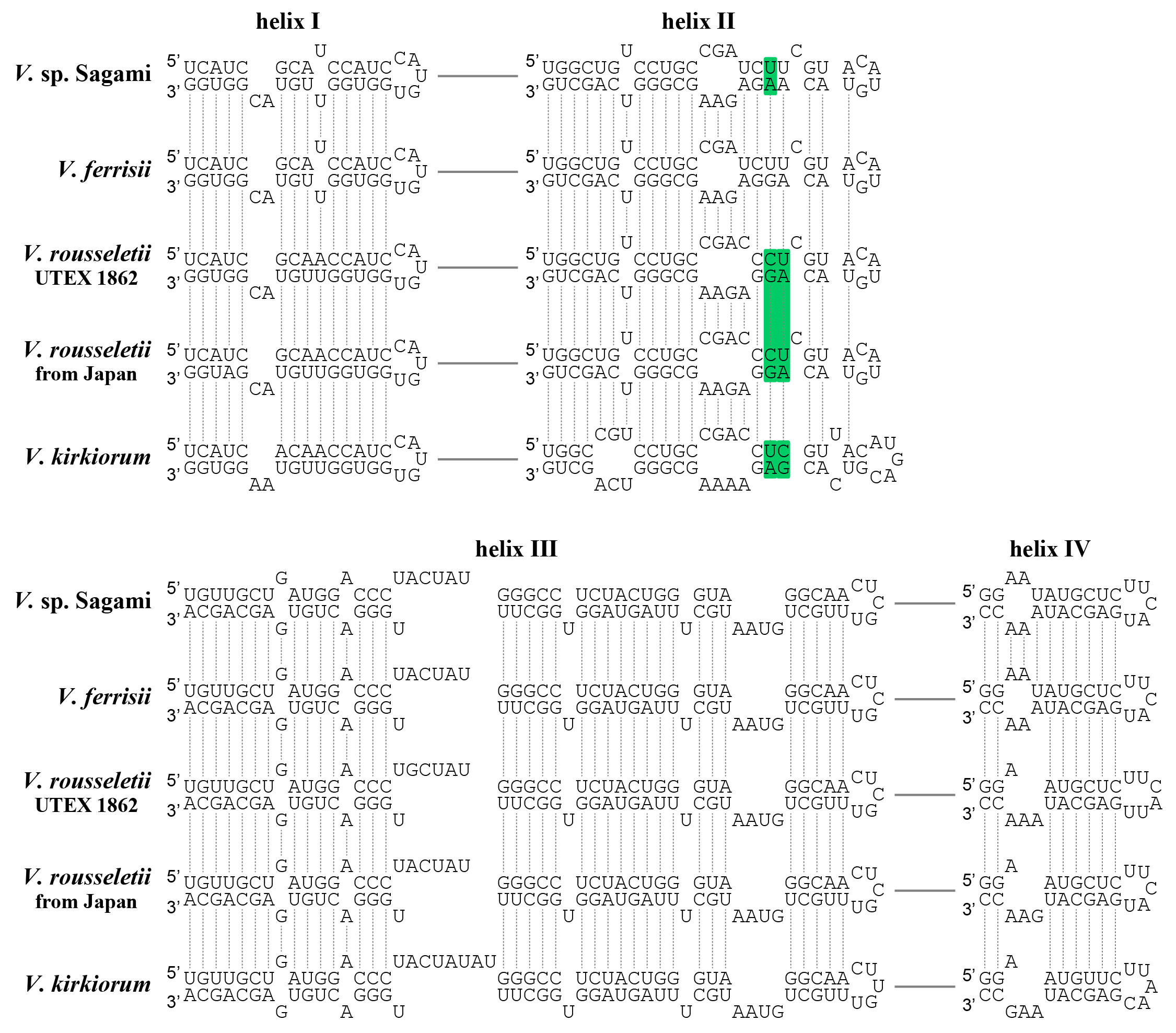


**S2 Fig. Comparison of helices of the secondary structure of nuclear ribosomal DNA internal transcribed spacer 2 transcripts between Volvox rousseletii from Japan and its related strain/species (Figs 4, 5).** Green backgrounds indicate compensatory base changes between *V. rousseletii* and the other species.
