# Supplementary material for: Morphological and molecular identification of the dioecious “African species Volvox rousseletii (Chlorophyceae) in the water column of a Japanese lake based on field-collected and cultured materials
Source: PLoS One. 2019 Aug 29;14(8):e0221632. doi: 10.1371/journal.pone.0221632 (PMC6715204; doi:10.1371/journal.pone.0221632)
Supplement: S1 Table — (DOCX) [file pone.0221632.s003.docx]

**S1 Table. Comparison of dioecious species originating from Japan and previously described dioecious species of *Volvox* sect. *Volvox.***

| Species | *Volvox rousseletii* from Japan | *Volvox rousseletii* from Africa ^a^ | *Volvox rousseletii* var. *lucknowensis* from India | *Volvox perglobator* | *Volvox prolificus* |
| --- | --- | --- | --- | --- | --- |
| Size of asexual spheroid (µm) | 331-423 x 352-476 | 690-2058 x 754-2180 | 610-629 x 544-766 ^b^ | 466-980 x 449-1100 | 400-1450 |
| Number of cells in asexual spheroid | 4700-11800 | 14000-42000 | 6000-8000 ^b^ | 2500-12000 | 9000–25000 |
| Number of gonidia in asexual spheroid | 4-8 | 1-16, usually 6-10 | 3-7 | 3-13 | 2–20, usually 6–12 |
| Size of male spheroid (µm) | 304-468 x 337-534 | 471-1443 x 559-1550 | 610-629 x 544-766 ^b^ | 315-400 x 320-440 | 140-1023 |
| Number of cells in male spheroid | 9100-17000 |  | 6000-8000 ^b^ | 4600-9200 | 5300-9200 |
| Number of sperm packets in sexual spheroid | 35-92 | 108-300 or more | 20-60 | 48-80 | 25-55, but up to 300 |
| Development of sperm packets in male sphered | Old male spheroid with sperm packets of early and final stages of development | Male spheroid with sperm packets of every stage of development | Male spheroid with sperm packets of all stages of development | Old male spheroid with only matured sperm packets | Old male spheroid with sperm packets of early stages of development |
| Size of female spheroid (µm) | 353-550 x 380-657 | 600-1464 x 644-1636 | 610-629 x 544-766 | 340-868 x 432-807 | 400-1090 |
| Number of cells in female spheroid | 8900-14000 |  | 6000-8000 ^b^ | 2200-12900 | 8500-1290 |
| Number of zygotes (eggs) in female spheroid | 52-97 | 60-227, sometimes up to 655 | 100-159 | 18-121 | 80-282 |
| Development of eggs/zygotes in female sphered | Old female spheroid with only matured zygotes | Old female spheroid with only matured zygotes |  | Old female spheroid with only matured zygotes | Old female spheroid with eggs and all stages of development of zygotes |
| Diameter of zygotes without spines (µm) | 31-39 | 35-44 | 32-33.5 | 21-34 | 30-35 |
| Shape of spines of zygote | Straight or slightly curved with acute apices | Curved or straight with acute apices | Broadly with conical apices | Straight with rounded apices | Straight with somewhat rounded apices |
| Length of spines  of zygotes (µm) | 3.4-6.4 | 4.5-12 | 3.7-5.5 | 2.5-5.4 | 5-9 |
| References | The present study (Figs 1-3) | Rich and Pocock [1], Smith [2], McCracken & Starr [3] | Iyengar [4], Smith [2], Iyengar & Desikachary [5] | Smith [2], Hanschen et al. [6] | Iyengar [4], Smith [2], Iyengar & Desikachary [5], Hanschen et al. [7] |

^a^ Including *V. rousseletii* f. *grinquaensis* Pocock.

^b^ Total measurement based on three types of spheroids.

**References**

1. Rich F, Pocock MA. Observations on the genus *Volvox* in Africa. Ann S Afr Mus. 1933; 16: 427–471, pls. 9–24.

2. Smith GM. A comparative study of the species of *Volvox*. Trans Am Microsc Soc. 1944; 63: 265–310.

3.. McCracken MD, Starr RC. Induction and development of reproductive cells in the K-32 strain of *Volvox rousseletii.* Arch Protistenkunde. 1970; 112: 262-282.

4. Iyengar MOP, Desikachary TV. Volvocales. New Delhi: Indian Council of Agricultural Research; 1981.

5. Iyengar MOP. Contributions to our knowledge of the colonial Volvocales of South India. J Linn Soc London Bot. 1933; 49: 323-373.

6. Hanschen ER, Davison DR, Ferris　PJ, Michod RE. On the rediscovery of *Volvox perglobator* (Volvocales, Chlorophyceae) and the evolution of outcrossing from self-fertilization. Evol Ecol Res. 2018; 19: 299–318.
