# Supplementary material for: Morphological and molecular identification of the dioecious “African species Volvox rousseletii (Chlorophyceae) in the water column of a Japanese lake based on field-collected and cultured materials
Source: PLoS One. 2019 Aug 29;14(8):e0221632. doi: 10.1371/journal.pone.0221632 (PMC6715204; doi:10.1371/journal.pone.0221632)
Supplement: Attachment — Submitted filename: Response to Reviewers.docx [file pone.0221632.s004.docx]

Dear Dr. Jim Umen,

Thank you very much for your positive review results of our manuscript entitled "Morphological and Molecular Identification of the Dioecious “African Species” *Volvox rousseletii* (Chlorophyceae) in the Water Column of a Japanese Lake Based on Field-collected and Cultured Materials" submitted for publication in ***PLOS ONE*** as a research article.

Based on the comments raised by Reviewr#1 and the academic editor, the manuscript has been revised completely. Accession numbers of the new sequences, study ID of the rDNA ITS regions and *rbcL*-*psbC* sequences used for construction of the phylogenetic tress (Figs 4, 5) and NIES strain designations of the new Japanese strains of *Volvox rousseletii* have been obtained and described in the revised manuscript.

Our responses to the comments by the academic editor and Reviewer#1 have been described in red below.

**********************

PONE-D-19-17716
Morphological and Molecular Identification of the Dioecious “African Species” Volvox rousseletii (Chlorophyceae) in the Water Column of a Japanese Lake Based on Field-collected and Cultured Materials
PLOS ONE

Dear Dr. Nozaki,

Thank you for submitting your manuscript to PLOS ONE. After careful consideration, we feel that it has merit but does not fully meet PLOS ONE’s publication criteria as it currently stands. Therefore, we invite you to submit a revised version of the manuscript that addresses the points raised during the review process.

I agree with Reviewer 1 about the high quality and overall suitability of this manuscript for publication.  In your revision and rebuttal please respond to the critiques of Reviewer 1 with whom I also agree regarding the last section where the discussion of Spirogyra and Chloromonas seemed out of place, and the need for details of the KOD polymerase method to be elaborated in the Methods section.

**Responses: Based on the comments, the conclusion section has been drastically revised by deleting the discussion of *Spirogyra* and *Chloromonas.* Detailed methods of PCR with KOD polymerase have been described in Materials and Methods section of the revised manuscript.**

One more minor point. In Fig. 1B there are asterisk symbols which are not mentioned in the legend.

**Response: The asterisk symbols have been specified in the legend for Fig. 1B in the revised manuscript.**

We would appreciate receiving your revised manuscript by Aug 30 2019 11:59PM. When you are ready to submit your revision, log on to [https://www.editorialmanager.com/pone/](https://www.editorialmanager.com/pone/) and select the 'Submissions Needing Revision' folder to locate your manuscript file.

If you would like to make changes to your financial disclosure, please include your updated statement in your cover letter.

To enhance the reproducibility of your results, we recommend that if applicable you deposit your laboratory protocols in [protocols.io](http://protocols.io/), where a protocol can be assigned its own identifier (DOI) such that it can be cited independently in the future. For instructions see: [http://journals.plos.org/plosone/s/submission-guidelines#loc-laboratory-protocols](http://journals.plos.org/plosone/s/submission-guidelines#loc-laboratory-protocols)

Please include the following items when submitting your revised manuscript:

- A rebuttal letter that responds to each point raised by the academic editor and reviewer(s). This letter should be uploaded as separate file and labeled 'Response to Reviewers'.
- A marked-up copy of your manuscript that highlights changes made to the original version. This file should be uploaded as separate file and labeled 'Revised Manuscript with Track Changes'.
- An unmarked version of your revised paper without tracked changes. This file should be uploaded as separate file and labeled 'Manuscript'.

Please note while forming your response, if your article is accepted, you may have the opportunity to make the peer review history publicly available. The record will include editor decision letters (with reviews) and your responses to reviewer comments. If eligible, we will contact you to opt in or out.

We look forward to receiving your revised manuscript.

Kind regards,

James G. Umen, Ph. D.
Academic Editor
PLOS ONE

Journal requirements:

When submitting your revision, we need you to address these additional requirements.

1. Please ensure that your manuscript meets PLOS ONE's style requirements, including those for file naming. The PLOS ONE style templates can be found at
   [http://www.journals.plos.org/plosone/s/file?id=wjVg/PLOSOne_formatting_sample_main_body.pdf](http://www.journals.plos.org/plosone/s/file?id=wjVg/PLOSOne_formatting_sample_main_body.pdf) and [http://www.journals.plos.org/plosone/s/file?id=ba62/PLOSOne_formatting_sample_title_authors_affiliations.pdf](http://www.journals.plos.org/plosone/s/file?id=ba62/PLOSOne_formatting_sample_title_authors_affiliations.pdf)

**Response: We have ensured that our revised manuscript meets PLOS ONE's style requirements**

2. We note that you have stated that you will provide repository information for your data at acceptance. Should your manuscript be accepted for publication, we will hold it until you provide the relevant accession numbers or DOIs necessary to access your data. If you wish to make changes to your Data Availability statement, please describe these changes in your cover letter and we will update your Data Availability statement to reflect the information you provide.

3. Thank you for stating the following in the Acknowledgments Section of your manuscript: "Funding: HN was supported by a Grant-in-Aid for Scientific Research (grant number 16H02518) from the Ministry of Education, Culture, Sports, Science and Technology (MEXT)/Japan Society for the Promotion of Science (JSPS) KAKENHI ([https://www.jsps.go.jp/english/e-grants/](https://www.jsps.go.jp/english/e-grants/)). The funder had no role in study design, data collection and analysis, decision to publish, or preparation of the manuscript."
We note that you have provided funding information that is not currently declared in your Funding Statement. However, funding information should not appear in the Acknowledgments section or other areas of your manuscript. We will only publish funding information present in the Funding Statement section of the online submission form.

Please remove any funding-related text from the manuscript and let us know how you would like to update your Funding Statement. Currently, your Funding Statement reads as follows:  "The funders had no role in study design, data collection and analysis, decision to publish, or preparation of the manuscript."

**Responses: I have removed any funding-related text from the revised manuscript. We would not like to update our Funding Statement "The funders had no role in study design, data collection and analysis, decision to publish, or preparation of the manuscript."**

4. Please include captions for your Supporting Information files at the end of your manuscript, and update any in-text citations to match accordingly. Please see our Supporting Information guidelines for more information: [http://journals.plos.org/plosone/s/supporting-information](http://journals.plos.org/plosone/s/supporting-information).

**Responses: I have included captions for our Supporting Information files at the end of our revised manuscript and updated any in-text citations to match accordingly. I have seen your Supporting Information guidelines**

Additional Editor Comments (if provided):


[Note: HTML markup is below. Please do not edit.]

Reviewers' comments:

Reviewer's Responses to Questions

**Comments to the Author**

1. Is the manuscript technically sound, and do the data support the conclusions?

The manuscript must describe a technically sound piece of scientific research with data that supports the conclusions. Experiments must have been conducted rigorously, with appropriate controls, replication, and sample sizes. The conclusions must be drawn appropriately based on the data presented. 

Reviewer #1: Yes

2. Has the statistical analysis been performed appropriately and rigorously? 

Reviewer #1: Yes

3. Have the authors made all data underlying the findings in their manuscript fully available?

The [PLOS Data policy](http://www.plosone.org/static/policies.action#sharing) requires authors to make all data underlying the findings described in their manuscript fully available without restriction, with rare exception (please refer to the Data Availability Statement in the manuscript PDF file). The data should be provided as part of the manuscript or its supporting information, or deposited to a public repository. For example, in addition to summary statistics, the data points behind means, medians and variance measures should be available. If there are restrictions on publicly sharing data—e.g. participant privacy or use of data from a third party—those must be specified.

Reviewer #1: Yes

4. Is the manuscript presented in an intelligible fashion and written in standard English?

PLOS ONE does not copyedit accepted manuscripts, so the language in submitted articles must be clear, correct, and unambiguous. Any typographical or grammatical errors should be corrected at revision, so please note any specific errors here.

Reviewer #1: Yes

5. Review Comments to the Author

Please use the space provided to explain your answers to the questions above. You may also include additional comments for the author, including concerns about dual publication, research ethics, or publication ethics. (Please upload your review as an attachment if it exceeds 20,000 characters)

Reviewer #1: This manuscript details the occurrence of Volvox rousseletii in Japan, the first time this species has been recorded outside of Africa. The manuscript is well written and is both concise and clear. The data presented is micrographs of laboratory cultivated asexual colonies, field collected sexual colonies, and phylogenetic analyses deriving from both asexual and sexual colonies (confirming that the sexual colonies do indeed correspond to the associated asexual strains). Overall the analyses are appropriate, the data recording is meticulous, the micrographs are excellent, and the paper should be published.

I have one major comment which should be addressed before publication. While the manuscript title, Abstract, and Introduction emphasizes the characterization of a species thought to be restricted to Africa, the Conclusions section instead emphasizes the challenge of characterizing sexual isolates in culture in Spirogyra and Chloromonas, other algae not previously mentioned. The Conclusions should be revised to address the novelty of this paper, the isolation of V. rousseletii in Japan as emphasized by the title, Abstract, and Introduction. The revised species range for V. rousseletii and availability of fresh cultures is worthwhile and should be emphasized. Similarly, the usage of KOD polymerase, an important methodological detail, should be moved to the Methods.

**Responses: Based on the comments, the conclusion section has been drastically revised by deleting the discussion of the challenge of characterizing sexual isolates in culture in *Spirogyra* and *Chloromonas*. Revised species range for *V. rousseletii* and availability of fresh cultures from Japan have been discussed in the conclusion section of the revised manuscript. Detailed methods of PCR with KOD polymerase have been described in Materials and Methods section of the revised manuscript.**

A few minor comments:

1. Hanschen et al 2018 Evol Eco Res published the psbC and rbcL sequences of Volvox perglobator (accession numbers KY489659 and KY489662), these should be included in Figure 5.

**Responses: Based on the comment, the *psb*C and *rbc*L sequences *Volvox perglobator* (accession numbers KY489659 and KY489662) have been included in Figure 5 of the revised manuscript.**

2. Page 5, last paragraph “Volvox” should be italicized when introducing VTAC media

**Response: Done as suggested.**

3. The implementation of maximum parsimony for phylogenetic analyses is outdated and should be replaced by Bayesian analyses. This analysis can be implemented in MrBayes.

**Response: The maximum parsimony has been replaced by Bayesian analyses using MrBayes in Figures 4 and 5 of the revised manuscript.**

4. Page 9, please do not abbreviate CBCs.

**Response: Done as suggested.**

5. Figure 1, the (*) and (b) are not specified in the legend for panels B and C.

**Response: The (*) and (b) have been specified in the legend for panels B and C in the revised manuscript.**
